# We are what we smell: the smell of dis-ease during lockdown

**DOI:** 10.1057/s41286-022-00132-9

**Published:** 2022-08-04

**Authors:** Louisa Allen

**Affiliations:** Faculty of Education and Social Work, University of Auckland, Auckland, New Zealand

**Keywords:** Subjectivity, COVID-19, New materialism, Dis-ease, Smell, Smellwalks

## Abstract

How does the COVID-19 pandemic shape subjectivity? This paper is concerned with contributing to theorising subjectivity at an ontological level. It draws on a feminist new materialist understanding of subjectivity as an intra-active becoming of human-non-human matter that includes smell. Smellwalks are mobilised to apprehend how subjectivity is altered via restrictions around movement and social connection during lockdown. This sensory method recognises knowing is not simply a cognitive practice and that odour actively shapes understandings of ourselves and the world. The varying presence and absence of odours in and out of lockdown eventuate a re-arrangement of subjectivity which draws on Vannini’s (2020) notion of atmospheric dis-ease. Lockdown produces a subjectivity of dis-ease which generates changes in perception of self and others, as sources of potential viral contagion. Lockdown’s material conditions engender a ‘socially flattened’ and ‘suspended subjectivity’ as our ‘normal’ selves are experienced as being put on hold until the global crisis abates.

## Introduction

This paper addresses the question of how the COVID-19 pandemic shapes subjectivity. It asks how the emergence of COVID-19 changes who we are? Rather than concentrating on individual psyche, this question attunes to the world’s materiality (Barad 2007) and sensory experience of daily life in a pandemic (Bull et al. 2006). Within the social sciences there is an emerging body of scholarship attempting to understand the momentous changes the virus has wreaked on daily life (Lupton and Willis 2021, Thorpe et al. 2021, Kwok 2020, Will 2020, Anderson and Knee 2021, Trnka 2021). These include government mandated practices of lockdown involving shuttering industry and commerce, ‘work from home’ directives, quarantine, social distancing and mask-wearing (Henrickson 2020). This paper seeks an alternative mode of understanding the pandemic beyond its current media-saturated, highly political, medical, and statistically dominated characterisation. In an endeavour to *know the pandemic differently*, and what it *feels* like to live through, the paper draws on the sensory resource of smell. It employs a sensory methodology known as ‘smellwalks’ (McLean 2019; Henshaw 2014) to experiment with understanding the nature of human subjectivity and how it changes during a pandemic.

At the heart of this discussion, is an ontological concern with subjectivity. This conversation was initiated in the first issue of this journal by Annemarie Mol (2008) in her paper ‘I eat an Apple’. Mol (2008) asked, ‘Does my apple only start to have subjectivity once it has become a part of me, after I have digested it, or should we be widening the category of potential subjects in such a way that it comes to include apples, too?’ (p. 30). Here Mol utilises eating/taste as a metaphor for understanding subjectivity and questioning boundaries between human subjects and inanimate objects. The current paper is also interested in challenging subject/object relations in an exploration of subjectivity, but via *smell*. Sensory approaches to sociology already acknowledge the relationship between smell and identity. As Synnott (1991) explains, ‘it is said that ‘we are what we eat’—but it is also true that we are what we smell like: fragrant or foul, good or bad’ (p. 446). This work recognises odour as a constituent component of individual and group identity that designates social and moral status on the basis of how smells are socio-culturally understood. The current paper contributes to this analysis, by reconceptualising the relationship of bodies, odour and subjectivity with insights from feminist new materialism. While it also argues that we ‘are what we smell’, it views a change in odours engendered by the practice of lockdown as altering subjectivity at an *ontological* level.

To undertake this reconceptualization, I draw on empirical insights from smellwalks in a small coastal town I call Bayside,^1^ in Aotearoa-New Zealand. Smellwalks involve moving through a geographical location inhaling, recording, and reflecting on smells encountered (McLean 2018). As a methodology, smellwalks recognise that, ‘smell is not just a”sensation”, but instead is one element of our experiential system that summons us to the world so that both the world and ourselves are constituted through that experience’ (Riach and Warren 2015, p. 15). Smellwalks aimed to explore changes to subjectivity in and out of lockdown as perceived through smell. A new materialist understanding of subjectivity is drawn on here along with Vannini’s (2020) notion of atmospheric dis-ease to capture the affective atmosphere of the town. Vannini (2020) explains that, ‘whereas disease is sickness and disorder, dis-ease is a social malaise infecting the body public via atmospheric contagion’ (p. 269). Perceiving the same malaise via smell during lockdown in Bayside, I think with the concept of dis-ease to understand its affect on residents’ subjectivity.

This discussion offers an understanding of how the lived reality of the pandemic changes the ontology of subjectivity and how this can be perceived through smell. Through attention to changes in smell in and out of lockdown, I argue it is possible to perceive how the pandemic shifts our understanding of ourselves in relation to others and the world. Lockdown produces a subjectivity of dis-ease which eventuates changes in perception of the self and others, as sources of potential viral contagion. Engendered by material conditions such as confinement to home, social distancing and the cessation of commercial and industrial enterprise, subjectivity is rearranged. As such, subjectivity becomes ‘socially flattened’ as individuals physically distance and routine collective activities halt. The cumulative affect of these material changes is a sense of ‘suspended subjectivity’, where normal modes of living and working are experienced as being ‘put on hold’ until they are again deemed ‘safe’. For those who can afford it (Bianchetti et al. 2020), this state of disruption also invites a slower, quieter, cleaner and more sensorially present mode of living.

The paper begins by establishing how subjectivity is theorised and its relationship to smell. The aim here is to reveal the paper’s contribution to thinking about subjectivity during the pandemic in ways that extend existing sociological theorisations of olfaction and identity. Next, an explanation of smellwalk methodology is provided to reveal how this was operationalised and to contextualise the findings. In the ensuing section, I address the central question of how lockdown smells change who we are? This discussion explores different modes of subjectivity produced via the absence and presence of smells in and out of lockdown. Finally, the paper considers the theoretical contribution of these findings and what they imply about the affect of COVID-19 on subjectivity in this moment of global crisis.

## Theoretical starting points

Agential realist theory provides an alternative mode of understanding the global pandemic beyond individualized notions of ‘human agency’ (Fullagar and Pavlidis 2021). Much official discourse relating to the virus, posits it as a force which can be controlled by humans via physical distancing, mask wearing, handwashing and vaccination. However, the virus’s unprecedented spread and speed of new infections across the globe suggests otherwise (World Health Organisation 2020). Another feature of official discourses of COVID-19 is the dislocating affect of recounting indices of death and infection from the actual embodied experience of living through the crisis. New materialist thought de-privileges human agency, focusing instead on how assemblages of the animate and inanimate, including phenomena such as odour, mutually produce the world (Fox and Alldred 2015, p. 399). This anti-anthropocentric perspective ‘explores a new ethics of collaboration and cooperation that might be derived from a worldview that sees agency in all things: stones, litter, memories, air, neural connections, tumours, atoms, desires, hands, almonds, bees, beliefs and sunshine (Nicholls 2018, p. 102). Employing a new materialist paradigm in their analysis of the pandemic, Fullagar and Pavlidis (2021) note, it enables a grasp of the embodied and affective relations that produce this crisis as an assemblage of human-non-human forces. Not only does this offer a more expansive and embodied rendering of life in the pandemic. It also recognizes the potency and effects of COVID-19 as emergent within an assemblage of human-non-human matter. This framing helps explain the difficulty of stemming the virus by recognizing agency as distributed relationally, rather than an inherent property of humans (Coole and Frost 2010).

To elucidate a feminist materialist theorization of subjectivity and the contribution it makes to thought, I first delineate how sociologists have conceptualized identity and smell. Sociology recognizes odour as a significant and pervasive element of social life which is undervalued and under researched (Howes 1991; Classen et al. 1994; Synnott 1991). Smell is conceptualised as a physiological phenomenon that also plays an important, but often unnoticed role in our culture and social lives (Synnott 1991). Drawing on the insights of Keller (1908) who lost her sight and hearing as an infant, adults are seen to ‘emit a distinct person-scent, like a ‘smell-print’ which is unique to each individual like a fingerprint and which bloodhounds and other dogs can identify’ (Keller cited in Synnott 1991, p. 442). Smell-prints form a constituent component of individual and group identity that mediate social interactions and group norms. While odours themselves are understood as intrinsically meaningless, they are attributed moral and social significance with varying affects. For instance, they can act as a boundary-marker between social classes as when the poor/homeless are identified by an ‘unwashed’ smell. A smell-print can therefore act as a statement of who one is, or how one is perceived by others. Such thinking establishes a clear link between smell and identity whereby how one smells is an indicator of social status and mediator of social interaction. At an ontological level, smell and identity here are understood as discrete entities, with a separation perceived between the material presence of an odour and the socially constructed meaning attributed to it.

For feminist new materialists, subjectivity is a state of perpetual *becoming*. This view diverges from sociological conceptualisations of identity and has implications for its relationship to smell. An agential realist account posits that humans cannot be understood as independent entities, but rather as ‘beings in their differential becoming, particular material reconfigurings of the world with shifting boundaries and properties that stabilise and destabilise with specific material changes’ (Barad 2003, p. 818). This means that subjectivity, or how we understand ourselves in relation to others and the world (Weedon 1996) is not simply a consequence of discourses of identity relating to gender, ethnicity, class or physical ability for example. Nor is identity solely determined by characteristics of human anatomy such as chromosomes and their expression as skin colour, genitalia or ‘normatively’ working limbs. Instead, subjectivity comes into being through what Barad (2007) characterises as ‘the world’s iterative intra-activity—its performativity’ (p. 152). This conceptualisation recognises the non-human and material constitution of subjectivity and embraces all phenomena conventionally understood as existing outside the human body. Returning to Mol’s (2008) example above, this recognises the intra-active agency of objects such as ‘apples’ or in relation to the pandemic, ‘COVID-19 viral particles’. The boundaries of the body are understood as porous, fluid and messy so that subjectivity is constantly being (re)made contingent upon specific intra-acting phenomena present in any time and space. The ontology of subjectivity within a new materialist rendering therefore comprises human-non-human phenomena of the world—including odour.

Another important distinction in a new materialist configuration of subjectivity is its conceptualisation of the relationship between human-non-human phenomena. What subjectivity becomes, is not a product of prior independent entities such as socially constituted meanings of identity or smell particles. Rather, subjectivity is configured via specific agential intra-actions present in the world’s larger material arrangement (Barad 2007). Humans do not simply determine who they are, nor can they determine the identity of others. Instead, this is decided in the moment of intra-action with other human-non-human phenomena. This conceptualisation recognises that ‘non-human matter possesses agency by virtue of its ability to affect and be affected by others’ (Nicholls 2018, p. 103). Applying this conceptualisation to odour and subjectivity means the material presence (or absence) of odours are seen to constitute subjectivity at an ontological level. It is not that certain smells only signify particular characteristics of our identity (i.e. that we are ‘wealthy’ or ‘poor’) but that odour *makes* who we are at a material/atomic level. Put another way, we do not inhale smells that become an indistinguishable part of us. Instead, odour is acknowledged as an agential materiality which intra-actively constitutes our subjectivity via its atomic presence. This rendering of the world recognises subjectivity’s materiality and, in this instance, *olfactory* way it eventuates.

## Mobilising Smellwalks

New materialist scholars attempt to understand the world via modes that are not primarily cognitive or representational (Hickey-Moody et al. 2016; Lupton 2021; Fullagar 2020). While smellwalks are a form of sensory methodology used previously by researchers in urban design and planning (Henshaw 2014) and media, art and design (McLean 2019) they exhibit features appealing to new materialists. For instance, smellwalks are premised on an understanding that knowing is not simply a cognitive practice but rather the world can also be apprehended through an embodied sense of smell. As McLean (2018) notes, ‘to walk and sniff is to know, in an unexpectedly fine and detailed way’ (p. 509). It is not words or thoughts that are given precedence during smellwalks, but the sensation of smell. The current smellwalks were guided by the overarching question, ‘what does smell eventuate in understanding the experience of lockdown’? This question relies on an alternative form of knowing (sense-making) based on olfaction rather than visual observation and cognitive awareness. Another characteristic of smellwalks is that in the act of inhalation ‘boundaries of the body and world, subject and object, self and other, are technically speaking blurred’ (Rhys-Taylor 2018, p. 27). This results because of what Synnott (1991) describes as the ‘profound intimacy of olfaction’, ‘whereby the fact that one person is breathing and inhaling the emanations of another means two become one in an olfactory sense’ (p. 453). While Synnott’s (1991) theorisation of this relationship differs from new materialists, such understandings echo ideas about human entanglements with the material world and other humans who share it.

Having linked smellwalks to new materialist thought, I now turn to how they were operationalized. As this is not a methodology paper, I only provide enough detail to contextualize the findings discussed next.^2^ Given the novelty of this method, this will be insufficient for some readers and a more comprehensive description can be found in (Allen 2021). It is important to note this method was not performed under ‘normal’ empirical circumstances, because of restrictions around movement and connection with others engendered by the pandemic. For instance, smellwalks have typically been conducted in cities (Diaconu 2011; Henshaw and Bruce 2012; Perkins and McLean 2020) however New Zealand was in Level 3 lockdown during this study which meant venturing outside my own neighbourhood was prohibited. Similarly, physical contact with others outside my own household ‘bubble’ was not allowed rendering the research autoethnographic. This regulation meant group smellwalks were untenable and instead I was obliged to undertake ‘solo walks’ or what MMcLean (2018) calls a ‘smelfie’. The length of lockdown and pressures of working from home dictated the number of smellwalks that could be undertaken. This meant it was only feasible to perform 3 smellwalks during lockdown and therefore I decided to conduct an equivalent 3 once out of it. Lockdown restrictions subsequently dictated the method in terms of participants, number of walks and their geographical reach.

In addition, three pilot smellwalks were undertaken to test digital recording equipment and experiment with walking routes. As time and weather conditions can affect smells encountered (Perkins and McLean 2020) smellwalks were undertaken at different times of day and varying days of the week. Each began from my house and followed walking routes typically taken by residents through the high street, around the boat Marina or along the beachfront. To record their trajectory, I downloaded a walking app (‘Mapmywalk’ produced by UnderAmour) to my phone enabling me to store details such as distance, time and length for later analysis. Early walks involved the technique of ‘smell-catching’ (McLean 2019) where I simply collected smells I encountered as I walked. In later walks, when I was more familiar with the town’s ‘smellscape’ (Porteous 1985) I engaged in the practice of ‘smell-hunting’ (McLean 2019) where specific smells such as natural (e.g. grass) or synthetic (e.g. cooking fumes) were actively pursued. Smell-hunting enabled me to explore whether smells present during lockdown were still apparent during out of lockdown walks. Their absence or presence and sometimes altered character, as in the case of whether clay was smelt wet or dry, could then be considered in relation to the experience of lockdown and changes to subjectivity.

The act of smellwalking involved frequently taking large inhalations of air and stopping when I encountered a distinctive smell such as diesel from passing trucks, cigarette smoke, or Wisteria hanging over a fence. I would then document the smell, identifying its source, listing details about its character including its intensity and reflections about its relationship to the experience of lockdown. These ‘smellnotes’ (McLean 2019) were recorded using the Voicememos App on my phone and were supplemented by a digital photo taken in the area the smell occurred. While photos could not always show the smell source, such as the lingering odour of spilt but now dried beer on the pavement, these images served as a memory aid when reviewing the smellnotes. As nasal attention wanes after 45 min (Perkins and McLean 2020) most walks lasted between 40 min to just over an hour. On return from each smellwalk, I transcribed the smellnotes and placed them in a PowerPoint presentation linking them with associated photos. This practice provided a comprehensive and sequential account of each smellwalk making analysis between those in and out of lockdown easier. During the analysis phase I noted what the absence and presence of smells might eventuate for subjectivity and potential changes created by lockdown.

The material and social landscape of Bayside is integral to what smells are present (and absent) in this environment. Specific features of Bayside’s topography like being a volcanic region surrounded by water, influence what and how odours materialise. For this reason, I provide a brief snapshot of the material and social characteristics of the town. A dominating element of Bayside’s landscape is the dormant volcano which sits across the sea on its eastern side. Subsequently, Bayside is built on volcanic clay and this propagates native flora and fauna such as flax, Pohutakawa and Nikau trees. These trees emit a bush-type smell, particularly after rain, distinctive from the fragrance of imported plants like Honeysuckle or Jasmine. Another prominent feature of the landscape is water, with a creek that runs through the town and empties at the beach’s northern end. Bayside is also perched on the edge of a 110-hectare freshwater lake formed by the explosion of a once live volcano. While Auckland is already renowned for its damp humidity, the presence of salt and fresh water lends the town an omnipresent ‘wet’ smell.

With a population of 5019, Bayside is a small town (Statistics New Zealand Tatauranga Aotearoa 2020) whose residents are considered middle class according to the 2018 census. This designation is awarded on the basis that more residents earn in excess of $150,000 compared with 3.9% in the wider Auckland region (Statistics New Zealand Tatauranga Aotearoa 2020). This socio-economic status is reflected in the town’s main centre where commerce thrives, and a new luxury apartment block is being erected. There is also a bustling high-street of local business including cafes and restaurants and a shopping mall with over 50 retail stores. The town’s ethnic composition contains a higher proportion of residents born overseas (40.4%) compared to the national median (27.1%). These people are mostly Asian (24.3%) hailing from East Asia (Chinese, Korean, Japanese) and South East Asia (Filipino and Vietnamese). The community’s largest ethnic group comprises Pākehā/European (72.4%) along with Māori (4.2%), followed by Pasifika (1.2%) with the remaining (3.4%) ethnicities nominated ‘other’. These environmental and social features are integral to the manifestations of odour and subjectivity explored next.

## How do lockdown smells change who we are?

To begin this section I introduce Vannini’s (2020) notion of atmospheric dis-ease to reveal how it re-arranges subjectivity during lockdown. In a 40-day diary documenting the emotional and embodied dimensions of quarantine on a small island off British Columbia, Vannini (2020) describes an atmosphere of social malaise. He characterises this as a ‘dis-ease’ produced by a plethora of effects that disrupt the usual rhythms of island life. Describing these he writes, ‘quarantine is a geography of what doesn’t happen: of cancelled events of missed chances, of a shuttered consumer society, of shattered kinship. For everything which quarantine and self-isolation shut down, they open up new atmospheres of dis-ease’ (Vannini 2020, p. 270). This dis-ease spreads like an infection that affects the ‘body public’ via a loss of ease and comfort to daily life. Such a rupture materialises in the ability to walk in the middle of a road usually populated with cars, the absence of seaplanes flying overhead and fact no friends or acquaintances shake hands. The suspension of daily habits and rituals becomes an ordinary effect expressed in the ‘impulse not to touch anything anymore…in the manic, obsessive, compulsive modes of haptic attention to everything that our bodies feel and touch’ (Vannini 2020, p. 270). Smellwalks harness this intensified haptic attention through smell and attempt to redirect it towards researching the pandemic. Drawing on the idea that ‘we are what we smell’, attention to smell discloses that lockdown produced a subjectivity of ‘dis-ease’ and subsequently a ‘socially flattened’ and ‘suspended’ sense of self.

Atmospheric dis-ease could be detected in Bayside via the odour of other people. Prior to the pandemic I was only peripherally aware of the smell-print of others which did not typically incite feelings of anxiety and risk. Increased attention to odour emitted by humans can be attributed to smellwalk methodology which focuses on olfaction. However, it is also a likely consequence of intensified anxieties about viral-laden breath associated with spread of the virus (Thorpe et al. 2021). During the pandemic’s early stages, research about how far virus particles travel each time someone exhales or coughs were recurrently reported in the media (Bourouiba 2020). These findings were used to determine and justify social distancing regulations implemented by governments across the globe. The following smellnotes offer examples of detecting the smell of others, particularly during smellwalks in lockdown.

So a lady has just passed me with her dog ….. and ah one thing that I could smell was not the dog… but sort of like a human smell of breath really and oxygen uhm and it made me think, gosh neither of us had a mask on … I can see how Covid spreads so quickly if you can be like, we were probably more than 2 metres apart from each other and I could still smell, uhm her. I’m sure she could smell me too. (Smellwalk 1, in lockdown, smellnote 3)

I’m walking down Bayside Road now… and I’ve just passed two people and I could smell their breath as well. No wonder this Covid thing spreads so quickly. (Smellwalk 2, in lockdown, smellnote 5)

Someone just rode past me on a push bike and coughed. I’m trying not to freak [nervous laugh] they weren’t wearing a mask. (Smellwalk 3, in lockdown, smellnMcote 10)

I just crossed the … high street and …3 ladies walked passed me and I could smell one lady’s perfume which was an Estee Lauder one which I recognised. (Smellwalk 4, out of lockdown, smellnote 4)

The smell-print of other humans takes on new significance during the pandemic. As Vannini (2020) explains, ‘dis-ease is discomfort, anxiety, suspicion, and fear of the unknown, of contamination, of the Other’ (p. 270). In the context of lockdown, the ability to smell others signals danger that social distancing measures have been breached and indicates an increased risk of viral contagion. While the smell of perfume could be enjoyable prior to COVID-19, during the pandemic, it is a stark reminder of the moment of dis-ease in which we now live. Any smell trace of others, pleasant or otherwise, now produces a sense of ‘dis-ease’ that can generate anxiety and signal risk of infection. As Vannini (2020) puts it, ‘in the new ordinary moment of collective dis-ease, everyone is suspect’ (p. 271).

A subjectivity of dis-ease eventuates here via the smell of others. Inhaling other people’s smell-print within an atmosphere of dis-ease engenders a particular way of understanding ourselves and others as potential sources of viral contagion. This in turn, produces feelings of anxiety and risk which contribute to a pervasive atmosphere of social malaise. From an agential realist perspective, the smell-print of others alters subjectivity at an ontological level. Although they identify as sensory rather than new materialist scholars, Riach and Warren (2015) offer a description of this change that captures smell’s ontological effect.

In relation to smell, bodies excrete substances with more or less detectable odours, and so we routinely inhale and ingest particles of every-body and every-thing we come into proximity with. However, at the same time, smell captures this movement of fragments that are simultaneously (1) in our selves, but (2) not fully of our selves and yet (3) make our selves (Riach and Warren 2015, 794)

Smell particles work intra-actively with our sense of who we are in relation to others and the world. When smell particles of others are inhaled, they can generate feelings of fear and anxiety that intra-actively eventuate a subjectivity of dis-ease. However, as Vannini (2020) explains, this is not an individual phenomenon, but an affect of a larger social arrangement of collective malaise produced by the disruption of daily life in lockdown.

Another moment in which dis-ease is discernible during smellwalks occurs while I am recording a smellnote in the local mall’s carpark. I have entered the carpark because I am drawn by the extraordinary sight of there being only two cars. Struck by its emptiness and the sense of space this produces, I inhale deeply and instead of the usual traces of petrol, rotting food and discarded rubbish, I smell ‘nothing’. The only hint of scent is salt from the sea which has travelled with the wind from the nearby beach. As I finish taking pictures of the vacant lot, there is a loud metallic crash behind me as a lone car reverses into the shopping trolley stand. The following is the two-part smellnote I record during this incident.

I’m standing in the carpark now at the top of the Bayside shopping centre and this place is usually packed, like you can never get a park in here because there are not enough spaces [sound of car engine starting in the background] but as you will see from the photo there’s just unlimited spaces today. It feels very empty.

So about 3 seconds after I took a photo uhm…there was this terrible crash and uhm a woman in the car behind me was backing her car up and she hit the ‘Trolley Return Stand’. I went to check on her and she said, ‘it was a lack of concentration’. Virtually the whole bumper has come off quite cleanly. (Smellwalk 3, in lockdown, smellnote 16).

**Fig. 1:**
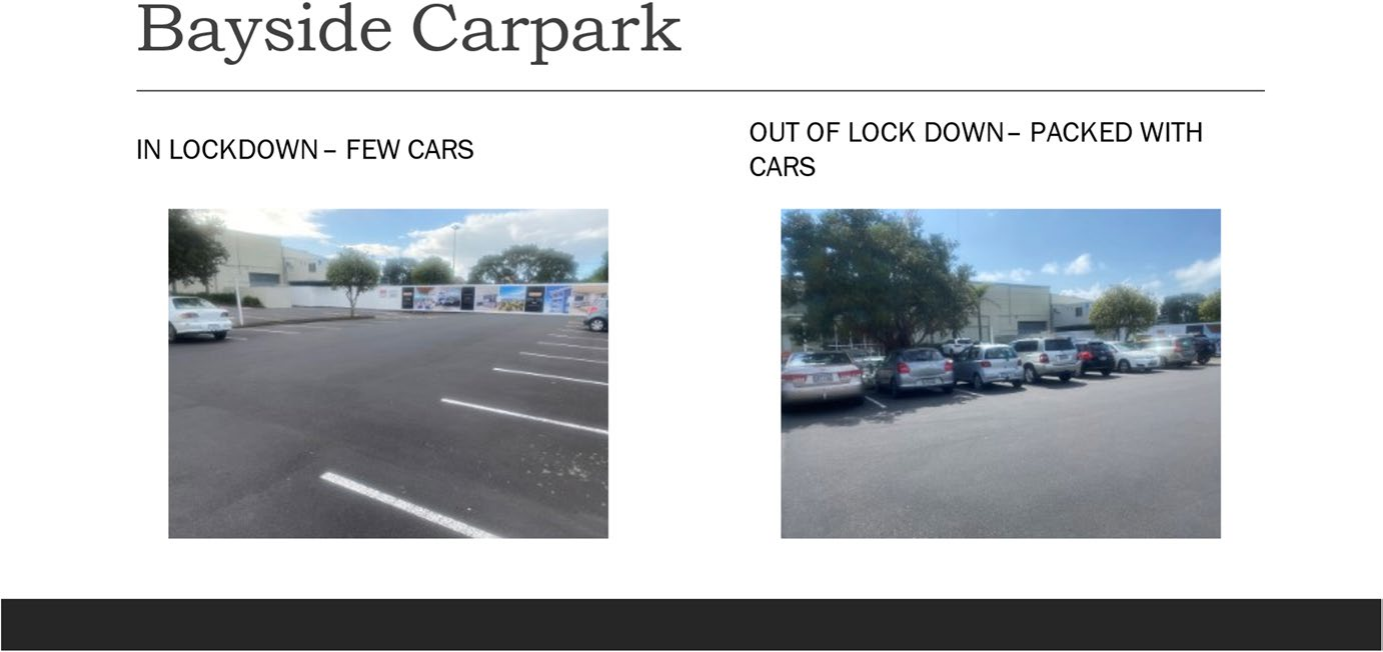
Comparison of Mall Carpark in and out of Lockdown

In her paper, ‘A whiff of Nothing: The Atmospheric Absence of Smell’, Stenslund (2015) draws on the work of Fowles (2010) to highlight how ‘absent stuff manages to impinge upon us just as much as present stuff’ (Fowles 2010, p. 25). The absence of normal smells such as petrol, rotting food and rubbish signal an atmosphere of dis-ease in Bayside. This sense of emptiness is accentuated by the visual absence of cars normally occupying this space (see Fig. 1). The feeling of dis-ease and sense of emptiness experienced in the moment of smelling ‘nothing’ is an affect which I suggest, is also felt by the woman in the car. On a normal day in the carpark packed with stationary and moving vehicles, people and trolleys, the likelihood of hitting something is far greater. However, so odd is the sight of this empty space and sense of dis-ease it generates, a lapse in concentration might be a typical response. The lockdown produces a subjectivity of dis-ease caused by the loss of ‘normal’ which inaugurates material affects like lack of concentration.

There were other moments during smellwalks when ‘the smell of nothing’ signalled atmospheric disease. These smell events occurred during lockdown walks, but their significance was not discernable until normal town activities resumed. The absence of smell was most apparent on the main high street and inside the shopping mall as captured in the following smellnotes.

I’m walking up the high street now and what I can notice, it’s just about dinner time and I can’t actually smell any sort of smell of cooking like from the restaurants and I guess that’s because most of the restaurants are closed. So that is definitely a difference I notice from non-Covid walks. (Smellwalk 1, in lockdown, smellnote 5).

**Fig. 2:**
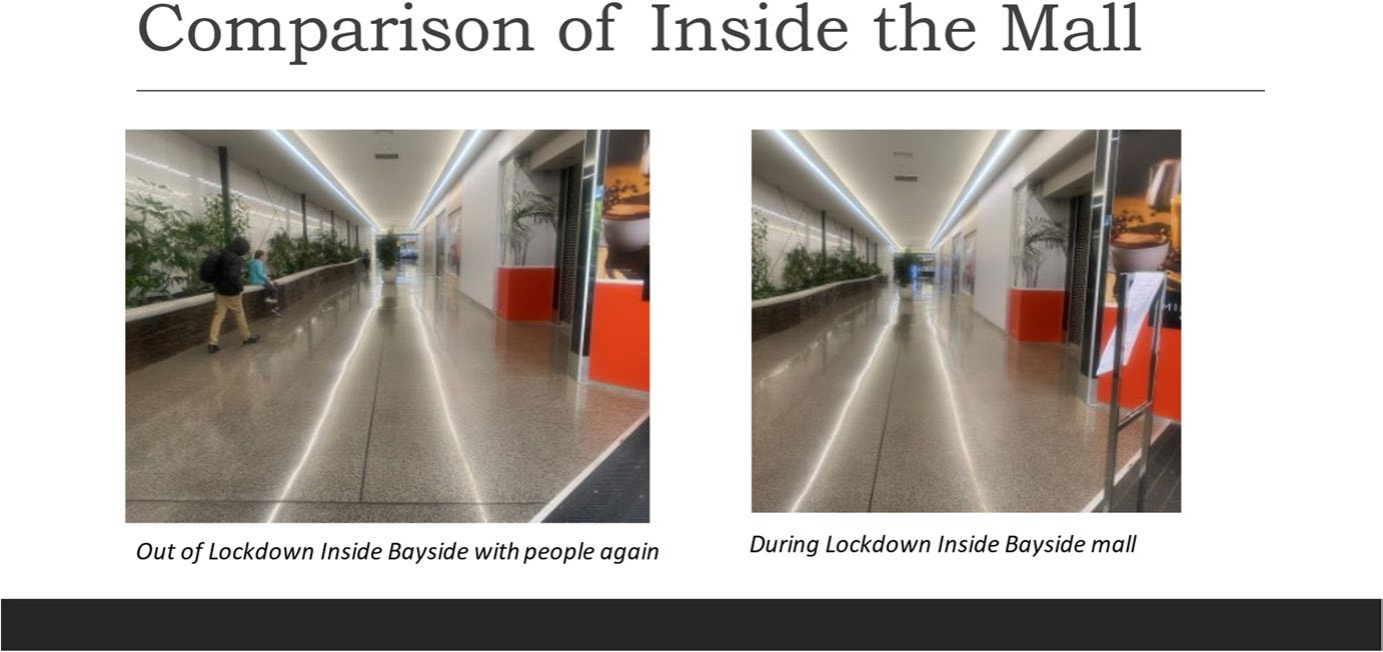
Comparison of inside the mall in and out of Lockdown

I’m just in the shopping mall now, just in the entrance way…It smells very shopping mall ‘y’ like cleaning products and slight warm air, compared to the air outside. It’s very empty, I’ve taken a photo. There’s hardly anyone there, it’s like [the movie] *I am Legend*. You can only access the Pharmacy through [that] entrance [I am standing in] at the moment. Normally people would be coming in and out of there to go into all parts of the mall. (Smellwalk 3, in lockdown, smellnote 13). [See Figure 2]

During out of lockdown walks I followed the same route up the main street and into the mall to compare smells now social distancing restrictions had lifted. The return of people and routine activities created a plethora of hot food smells like the yeasty smell of bread from the bakery. The smellnotes below convey my delight in reacquainting with these smells as they signal a return to ‘normal’ life and a way of living that had been suspended during lockdown. The next three smellnotes occur as I meander up the main street noting smells emanating from shop doorways.

**Fig. 3:**
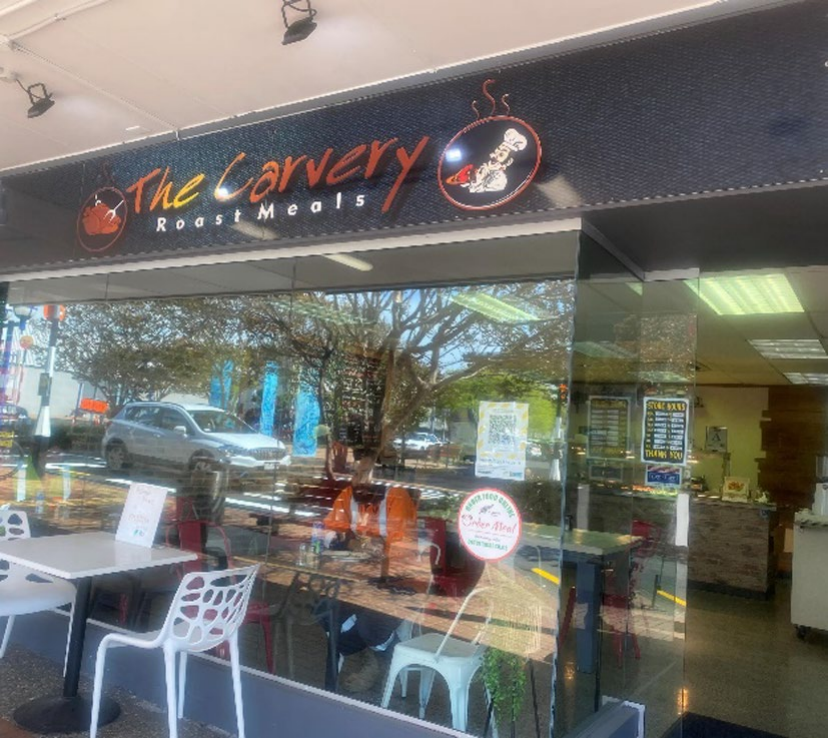
The smell of hot cooking food from the carvery

So I am now outside of the Carvery Roast shop and it smells divine, like meat and gravy. It shows you how much I have missed normal smells given that I rarely eat meat! [Figure 3]

Now I’m going passed the Baker’s Delight which also smells divine like bread.

Just been passed the Kebab shop [deep sniff] that smells gorgeous too [sniff] like lamb and onions. Those are pretty strong smells emanating from the high street. (Smellwalk 3, out of lockdown, smellnotes 5, 6 and 7)

**Fig. 4:**
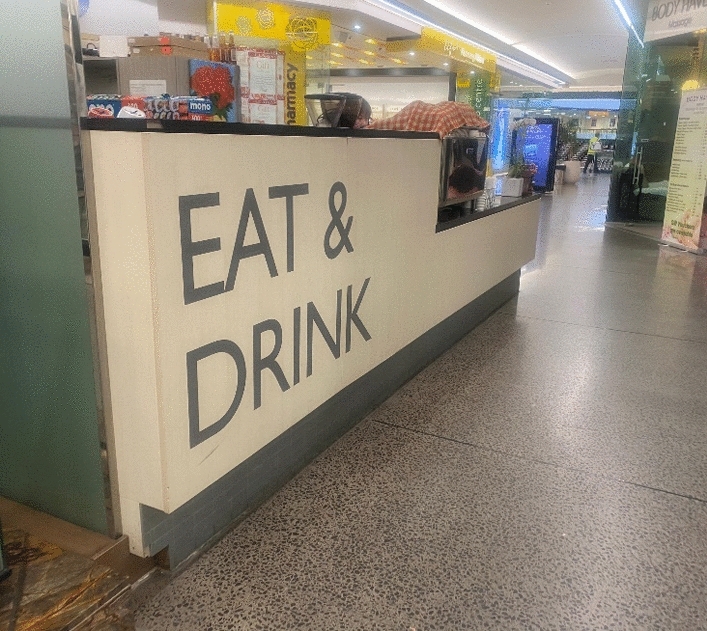
The smell of coffee and perfume from inside the mall

The following smellnote is taken as I enter the shopping centre for the first time in six weeks (See Fig. 4).

Right I’ve just come up the escalator in the mall uhm and I have gone passed the ‘Expresso Café’ and it smells of coffee. You can hear quite a lot of ah uhm noise in the mall at the moment, it’s pretty busy, lots of people are about. When I passed the pharmacy it smelt of perfumes and uhm medicine and things. Uhm and I’m now going passed Sportscraft which always has the most amazing sort of smell attached to it. Like kind of uhm ah, perfume, a perfumey smell that’s really distinct with  Sportscraft. (Smellwalk 5, out of lockdown, smellnote 7)

Smell researchers typically attribute the smell of ‘nothing’ to a process of habituation which occurs when an odour is so familiar and ubiquitous it is no longer noticed (Henshaw 2014; Porteous 1985). For example, when grass and animal odours which permeate rural landscapes are indiscernible or ignored, by farmers. However, I suggest not being able to detect odour is a consequence of the cessation of routine movement and activities in Bayside. The ‘smell of nothing’ indexes the loss of normal life in the town and a middle-class existence which involves eating at restaurants and meeting friends for coffee. How one normally understands themselves as a consumer and socially engaged citizen, produced through activities such as shopping at the mall and meeting friends, is lost through lockdown restrictions. Kalaichandran (2020) describes the impact of moments of crises such as the Sri Lankan civil war which her parents escaped, in terms of two forms of ‘loss’.

According to my mother, there are two unique forms of grief that everyone touched by war understands. There’s the grief associated with the loss of human life—through bombings and brutal combat, and through the disease that runs rampant when health care and all other social services are halted. Then there’s the grief associated with the loss of a life as we once knew it: loss of country, loss of employment, loss of identity as a ‘prewar person’, and the subsequent need to start over. The two run along together like two dark snakes intertwined. (Kalaichandran 2020, p. 1)

The odour of nothing constitutes a subjectivity of dis-ease and ‘marks the loss of life’ and ‘loss of identity’ as Kalaichandran (2020) characterizes it, in Bayside prior to the Covid-19 pandemic. When smelling nothing, I experience the emptiness the town exudes caused by the shuttering of business and cessation of routine activities. At a theoretical level the absence of odour *makes* subjectivity as much as its presence, because from a material perspective smell is never absent. As Stenslund (2015) explains, ‘materially, smell is omnipresent yet invisible and intangible, and despite its molecular presence it can appear as presence or absence nevertheless’ (p. 345). While I detect ‘no smell’ often during smellwalks, smell particles are always present engendering a subjectivity of loss for a way of life now missing.

While sometimes I could literally smell ‘nothing’, at other times I failed to detect familiar odours I was expecting. For example, during lockdown walks the aroma of coffee which typically emanates from high street cafés was missing. When such anticipated smells failed to materialize, I interpreted this as ‘no smell’, until I began to attune my olfactory senses more acutely. It became apparent that as synthetic smells like petrol receded from the town, more organic odours emanating from the natural environment emerged. These organic smells were most noticeable during the last two walks following six weeks of lockdown when my olfactory skills were honed. What had become noticeable was Bayside’s pervasive ‘wet’ smell which vacillated between the stagnant smell of the local creek and salt-tang of the sea.

I’m coming down to Bayside creek now and as I’m approaching it I can smell more wetness [inhale loudly]. I can just smell water, not the water like at the beach, which has got a salt taste to it but sort of more kind of stagnant water. And now actually……I can smell one of those onion plants that grow wild. I can’t actually see them though…. I’m going to take a photo. (Smellwalk 3, out of lockdown, smellnote 6).

Naturally occurring vegetation like ‘onion weed’ and smell of grass and native trees became discernible even when these couldn’t be immediately sighted. Similarly, the dusty sulphur scent of clay from neighbourhood gardens caused by an unseasonably dry autumn also surfaced. Out of lockdown, this clay exhibited a stronger and acrid odour coinciding with the resumption of the local building industry and its mass excavation of wet clay. The emergence of more natural environmental odours may be attributable to a reduction in anthropogenic emissions caused by the interruption to road and airline transportation during lockdown documented by other researchers (Verisk 2020). As Asumadu Sarkodie and Asantewaa Owusu (2021) note, ‘social distancing policies instituted across countries are reported to have yielded environmental sustainability. Total lockdown in many countries saw a halt in carbon and energy intensive economic sections such as manufacturing and transportation’ (p. 5007). In Bayside, this was evidenced in the town air appearing clearer, intensifying the blue of the sky and rendering the volcano’s outline more vivid. This state of disruption invited a slower mode of living where the world was quieter and environmentally cleaner. A more sensorially present subjectivity, attuned to the material and natural world could emerge. As Bianchetti et al. (2020) observe, ‘quarantine can be a luxury, for those who can afford it—an opportunity to reclaim some personal time and even a condition to aestheticize the emptiness of cities as a dystopian object of contemplation…But for those who experience poverty, marginalization, risks of violence, and housing discomfort, there is no spiritual landscape in the ruins’ (p. 302).

This loss of normal life during lockdown eventuates a re-arrangement of subjectivity that *leaves us feeling as if our normal selves have been ‘put on hold’*. To be clear, this is not a literal halting of subjectivity in any conventional temporal sense, as within a new materialist conceptualisation *who we are is always becoming*. It is a feeling however, that has an ontological basis as it eventuates in the moment of intra-action with other elements of the pandemic assemblage, such as the presence or absence of specific smells. The notion of ‘suspended subjectivity’ is subsequently conceptualised within a new materialist paradigm as an iterative becoming, contingent upon other elements of the pandemic assemblage being present, e.g. closure of businesses, lockdowns, quarantine and social distancing measures.

**Fig. 5:**
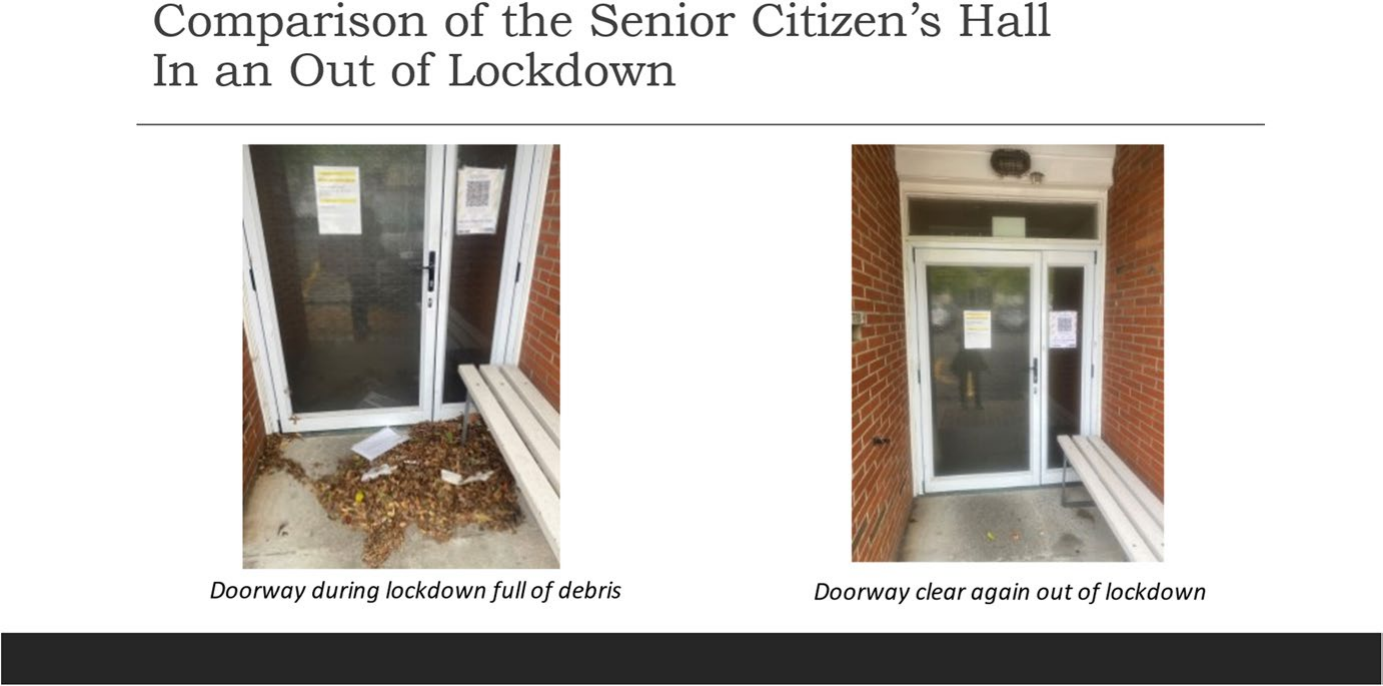
Comparison of Senior Citizens Hall in and out of Lockdown

What makes this experience of ‘normal self’ feel ‘suspended’ rather than ‘ended’, is an anticipated return to pre-COVID-19 life once lockdown finishes. This sense of suspension is materially conveyed via postponed events, waiting for alert levels to lower, borders to open, rules around people gathering to loosen and retail to reopen. A subjectivity where the normal self is experienced as suspended, is evident when comparing images and smellnotes relating to the local Senior Citizens hall. A photo of the hall doorway during lockdown reveals it has been shut for many weeks as a pile of leaves and debris gather around the normally immaculate entranceway (see Fig. 5). This area smells of dry leaves, dust and a slightly stale odour of rubbish. When I pass the hall again out of lockdown the entranceway is once again pristine indicating its return to use for a myriad of activities including Karate, a holistic market and Zumba classes. On the last smellwalk the door is open and I hear jaunty music as a group of senior citizens dance with partners. Peering inside, I catch a whiff of the musty air indicative of the halls’ prolonged closure and a hint of tea and scones for morning tea. The ‘socially flattened subjectivity’ of lockdown is replaced with the buoyant presence of senior citizens dancing and enjoying each other’s company again. The suspension of a social self during lockdown is replaced by re-connection with others, but also the lingering thought that *it might happen again*.

## Closing comments

How subjectivity is changed by COVID-19 has been the focus of this paper. Exploring this question seeks to contribute to theoretical conceptualizations of subjectivity by employing feminist new materialist thought. Given one of the symptoms of COVID-19 can be a loss of smell, it is perhaps appropriate this research mobilizes this sense to understand the pandemic’s affect on subjectivity. As the saying goes, we often do not fully realize the value of something until it disappears. Employing smellwalks to understand the experience of living through this global crisis and what it *feels* like, rather than what it does (infect and kill) is *political*. In a time when breathing is made difficult for those infected with the virus and the act of breathing freely (without a mask and in public spaces with unknown others) is a luxury, the operationalization of smellwalks is an exercise of agency. What this paper suggests, is that via smell, we can *know* the pandemic differently in a mode that provides sensory insights about how we understand ourselves in relation to others and the world.

The paper argues *we are what we smell*. This builds on sociological understandings of smell’s relationship to identity where odour bestows social status and regulates interaction. In this conceptualisation, although odours enter the body and become an integral and indistinguishable part of individual identity, they are understood as pre-existing and distinct entities. Within a new materialist rendering, subjectivity comes into being via iterative intra-activity and neither odour particles nor humans are recognised as separate or discrete phenomena. Subjectivity subsequently becomes an entanglement of human-non-human phenomena present in any time and space. This theorisation acknowledges the agency of non-human things like odour, to actively constitute subjectivity at an ontological level. The advantage of such a conceptualisation for understanding the pandemic, is it helps explain why human endeavours to prevent the virus have been so challenging. When agency is perceived as distributed relationally and COVID-19 an assemblage of human-non-human forces, it is apparent humans do not hold exclusive power to eradicate the virus.

By paying attention to the presence and absence of smells during smellwalks it is possible to apprehend how COVID-19 changes subjectivity. Alertness to the smell of others eventuates a subjectivity of dis-ease. Dis-ease is ‘…a social malaise infecting the body public via atmospheric contagion’ which Vannini (2020) observes in a small island community off British Columbia (p. 269). Such atmospheric dis-ease intra-actively re-arranges subjectivity. This process occurs when smell particles of others are inhaled inciting anxiety and fear of risk and producing a subjectivity of dis-ease. A subjectivity of dis-ease changes perceptions of self and others, as sources of potential viral contagion. This in turn has material affects, like avoiding physical closeness with others and an intensified haptic attention to every-thing as potentially infecting.

Atmospheric dis-ease has other outcomes for subjectivity. The material consequence of disruption to normal community activities during lockdown produces subjectivity as ‘socially flattened’. Habitual ways of being, like meeting up with friends, eating in restaurants, and enjoying dance classes, cease to occur and flatten social engagement. This change to subjectivity is discernible via the absence of smells typically present in the town such as coffee and hot food. They are replaced by an ‘odour of nothing’ that marks a loss of way of life and identity prior to COVID-19. However, a dissipation of synthetic smells in the town enables more organic and natural odours to emerge. For those who can afford it, lockdown presents a moment of sensorially present subjectivity where quietness and a new clarity to the natural environment can be appreciated. These materializations culminate in a ‘re-arrangement of subjectivity’ where who we understand ourselves to be, feels as if it has been put on hold until lockdown ends and our usual lives resume. However, within a new materialist understanding of subjectivity, any re-turn always traces the past, eventuating a new iteration of self, perpetually marked by COVID-19.

## Footnotes

1 To preserve some semblance of privacy ‘Bayside’ is a pseudonym. Similarly, there are a limited number of photographs included of the town to reduce the possibility of its identification.

2 For instance, a reflective account of my role as a smell researcher during the pandemic is missing here and can be found in the book, ‘Stories of the Pandemic’ (in press) edited by Lisa Moran and Kateřina Sidiropulu Jankuů (see Allen in press).
